# Placental growth factor (PlGF) and soluble fms-like tyrosine kinase-1 (sFlt1): powerful new tools to guide obstetric and medical care in pregnancy

**DOI:** 10.1177/1753495X251327462

**Published:** 2025-04-03

**Authors:** Rachel A Gladstone, John W Snelgrove, Kelsey McLaughlin, Sebastian R Hobson, Rory C Windrim, Nir Melamed, Michelle Hladunewich, Sascha Drewlo, John C Kingdom

**Affiliations:** 1 Division of Maternal-Fetal Medicine, Department of Obstetrics & Gynaecology, Mount Sinai Hospital, Canada; 2 Division of Maternal-Fetal Medicine, Department of Obstetrics & Gynaecology, Sunnybrook Health Sciences Centre, University of Toronto, Canada; 3 Department of Medicine, Sunnybrook Health Sciences Centre, University of Toronto, Canada; 4 Sunnybrook Research Institute, Sunnybrook Health Sciences Centre, University of Toronto, Canada

**Keywords:** PlGF, sFlt1, preeclampsia, placenta, pathology, obstetrics

Despite many early 21st century advances in the delivery of healthcare to pregnant patients, all providers remain fearful of the maternal and perinatal consequences arising from undiagnosed placental dysfunction. If not recognized in a timely fashion, severe placental disease may present as life-threatening severe preeclampsia, where new-onset hypertension is associated with functional impairment of critical maternal organs, including the brain, heart, liver, and kidneys.^1^ In tandem, progressive placental damage can cause fetal growth restriction (FGR) which when undiagnosed can lead to a potentially avoidable stillbirth.^2^ Evidence-based interventions currently exist to mitigate against these major adverse health outcomes, yet to be effective the underlying placental disease needs to be identified, especially among pregnant individuals with high-risk clinical profiles. In well-developed and equitable healthcare systems, up to 2% of pregnant individuals will require iatrogenic preterm delivery due to preeclampsia with severe features, a diagnosis that is established by *de-novo* severe hypertension with critical organ involvement,^3^ while up to one in 200 pregnancies will end as a stillbirth prior to labor.^4^ The placenta-mediated contribution to obstetric morbidity and mortality is slowly worsening over time, as several epidemiologic links to placental dysfunction, namely moderate obesity, advanced maternal age, use of assisted reproductive technologies, chronic hypertension and preexisting diabetes, all continue to rise in prevalence.^4^ Closer maternal surveillance of a subset of patients at most risk of preeclampsia with severe features could mitigate against life-threatening critical maternal organ injury via interdisciplinary care and planned iatrogenic preterm birth.^5^ Efforts to increase the general use of obstetric ultrasound examinations has had minimal effect on stillbirth rates; for example in Canada, a 50% increase in obstetric ultrasound examinations performed between 1996–2006 did not impact the rate of stillbirth.^6^ How, therefore, can we better address these major challenges presented by serious placental diseases?

## Placental growth factor (PlGF) and soluble fms-like tyrosine kinase-1 (sFlt1)

The solution may well be a greater role for two placenta-derived proteins, PlGF and sFlt1, that circulate in maternal blood, described as “angiogenic proteins” because they regulate many facets of arterial vascular function. Discovered in the human placenta in 1991,^7^ PlGF is highly expressed by the developing placental villi, and also systemically by the vascular endothelium.^8^ Maternal circulating PlGF levels vary considerably across gestational age and therefore require gestational age-specific interpretation^9^ (see Table 1); levels rise to a peak at 28–30 weeks of gestation, then decline towards term. The principal actions of PlGF are to promote local angiogenesis and augment the vasodilatory actions of vascular endothelial growth factor (VEGF). A progressive rise in circulating PlGF levels facilitates the decline in maternal systemic vascular resistance observed in normal pregnancy; as such, the large increase in blood volume, accompanied by a higher cardiac output has negligible net effect on blood pressure, and the pregnancy thus is supported to continue safely to term.^10^ sFlt1 is likewise synthesized by the syncytiotrophoblast surface of the placenta; levels rise slowly during the third trimester towards term. sFlt1 directly antagonizes the potent local paracrine actions of VEGF residing in the glycocalyx surface of the endothelium, where VEGF's principal action is to generate the vasodilator nitric oxide.^11^ Longitudinal changes in both PlGF and sFlt1 have been described^12,13^; since PlGF declines as sFlt1 continues to rise during the third trimester, the sFlt1/PlGF ratio progressively rises as pregnancy advances towards term.

**Table 1.** Gestational age-specific values for circulating maternal PlGF (pg/mL) at the 2.5th, 5th, 10th, and 50th centiles derived using quantile regression of merged data from comparable Cambridge and Toronto cohorts of Caucasian nulliparous patients (Elecsys platform, Roche Diagnostics, Penzberg, Germany).

Gestational age (week)	2.5th percentile	5th percentile	10th percentile	50th percentile
12	19	21	25	40
13	23	26	30	49
14	28	32	38	63
15	35	42	49	84
16	45	53	64	110
17	57	67	80	139
18	69	81	96	166
19	80	94	111	191
20	91	106	126	217
21	101	118	141	246
22	110	131	157	280
23	120	144	175	321
24	131	159	196	368
25	140	173	216	420
26	148	186	234	471
27	153	194	248	513
28	153	196	252	539
29	147	189	246	542
30	135	175	229	523
31	119	155	206	489
32	103	134	179	444
33	89	116	155	396
34	78	101	134	349
35	71	90	118	305
36	67	83	106	267

*Note*. PlGF, placental growth factor.

Source: Reproduced with permission from K. McLaughlin et al. Low-molecular-weight heparin in pregnancies at risk of placental dysfunction. Am J Obstet Gynecol. 2022.

## Placental pathology of low PlGF and high sFlt1

Placental pathology analysis following preterm delivery for preeclampsia with severe features or FGR most commonly reveals visible and microscopic features of a disease described as maternal vascular malperfusion (MVM).^14^ Our recent experience found MVM disease in 85% of periviable stillbirths associated with preeclampsia with severe features or FGR.^15^ *In vivo*, pregnancies destined to develop placental MVM disease have persistently low circulating PlGF levels and reduced uteroplacental perfusion in the early second trimester, identified bilaterally with notched high-resistance uterine artery Doppler waveforms.^16,17^ Just before delivery, magnetic resonance imaging of the placenta in pregnant individuals with preeclampsia confirms the chronically hypoxic state of the placenta.^18^

**Figure 1.**
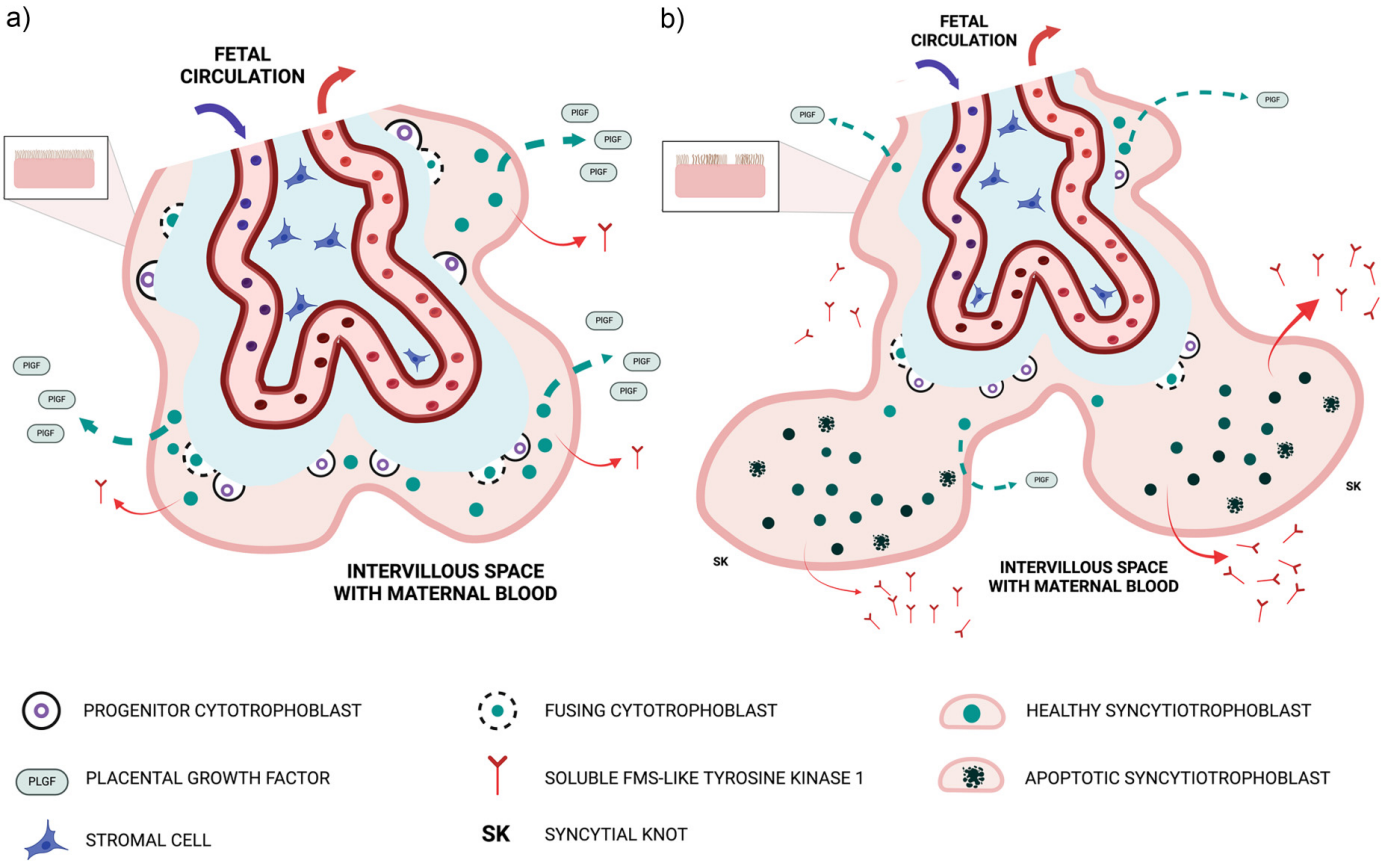
The placental basis of suppressed PlGF and hyper-secretion of sFlt-1.

The molecular basis of the dysregulated circulating angiogenic profile, comprising low PlGF and elevated sFlt1, resides within the placental villi and is illustrated in Figure 1. The placental villous surface in contact with maternal blood is a continuous, multinucleated structure termed the syncytiotrophoblast. This layer is generated by underlying progenitor villous cytotrophoblast cells, which divide asymmetrically to deliver “daughter” cells by fusion into the expanding outer syncytiotrophoblast in contact with maternal blood (Figure 1(a)). Syncytial fusion is a continuous process that drives the progressive synthesis and secretion of PlGF.^19^ Chronic underperfusion of the placental villi, beginning in the early second trimester, results in premature loss of progenitor cytotrophoblast cells.^20^ Syncytial fusion is therefore restricted; cytotrophoblast depletion combined with patchy areas of apoptosis and necrosis of the syncytiotrophoblast is found in placentas from preterm deliveries of pregnancies complicated by preeclampsia with severe features.^21,22^ Understandably, the capacity of the placenta to synthesize and release PlGF becomes severely diminished (Figure 1(b)). Restricted syncytial fusion also causes the outer syncytiotrophoblast to reorganize into syncytial knots,^23^ which become the principal sites of aberrant sFlt1 production.^24^ Explanted placental villi derived from pregnancies with preeclampsia with severe features condition their cultured media to show a high sFlt1/PlGF ratio, and this media is rendered highly anti-angiogenic *in vitro*.^25^ Serial maternal blood analysis of PlGF and sFlt1 levels in the early second trimester are consistent with the molecular pathology; PlGF levels are 50% lower in pregnancies destined to deliver preterm with preeclampsia, while sFlt1 levels are unaffected at this stage.^16^ It is only in the later stages of disease progression, typically at >24 weeks’ gestational age, that sFlt1 levels become elevated.^12,13^

## Maternal and fetal consequences of placental MVM disease

The combination of grossly visible features (small placenta with infarcts) and several microscopic features (diseased decidual spiral arterioles and syncytial knot formation) comprises the key diagnostic features of placental MVM disease. Collectively, these structural changes contrive to reduce PlGF output and impair fetal growth, especially when PlGF falls below 100 pg/mL.^18,26^

Pregnant individuals who develop preeclampsia with severe features before 37 weeks of gestation typically have circulating PlGF levels less than 100 pg/mL, which is well below the 5th percentile at all viable gestational ages up to 36 weeks.^9^ They also exhibit high levels of sFlt1, with higher ratio levels proportional to fetal and maternal disease severity (reviewed recently^27^). Hemodynamically, early-onset preeclampsia is characterized by high systemic vascular resistance and reduced cardiac output.^28–30^ The resultant impaired perfusion of critical organs ultimately leads to coagulopathy and HELLP syndrome, and may rarely result in neurologic complications including seizure or stroke, renal failure, or cardiac dysfunction.^31^ High sFlt1 levels, as opposed to low PlGF levels, appear to mediate maternal critical organ injury.^32^ In healthy individuals, the only known site of intense sFlt1 production is at the perimeter of the cornea, where sFLT1 blocks angiogenesis to thereby prevent corneal neovascularization.^33^ In contrast, high systemic levels of sFlt1 are toxic to critical organs; as an example, the commercially available monoclonal antibody bevacizumab used to limit some forms of metastatic cancer may cause posterior reversible encephalopathy syndrome (PRES),^34^ which is a recognized neurologic complication of preeclampsia. High circulating sFlt1 levels are also strongly associated with cardiac dysfunction including peripartum cardiomyopathy.^35,36^ In the kidney glomeruli, the podocyte cells, designed to prevent the escape of larger circulating proteins into urine, strongly express VEGF. High circulating levels of sFlt1 deprive these cells of VEGF signaling, such that they begin to detach or fragment into urine with the resultant glomerular damage causing proteinuria that is a hallmark of preeclampsia with severe features.^37^

## Utility of sFlt1 and PlGF for the diagnosis and prognosis of suspected preeclampsia

The above evidence has provided a strong foundation for studies that have evaluated either PlGF alone^15,38^ or the sFlt1/PlGF ratio^39,40^ to improve diagnostic accuracy of preeclampsia, demonstrating the superiority over traditional tests (such as proteinuria, elevated uric acid or liver enzymes) to exclude or make a diagnosis of preeclampsia before 36 weeks of gestation. With >98% negative predictive value, both PlGF (>100 pg/mL) or the sFlt1/PlGF ratio (≤38) demonstrate robust test performance.^15,38–40^ Consequently, both test methods have been endorsed by the UK National Institute for Clinical Excellence (NICE),^41^ the Ontario Ministry of Health in Canada,^42^ and the US Food and Drug Administration.^43^ The PARROT randomized control trial indicated that PlGF testing improves time to diagnosis of preeclampsia,^31^ while the subsequent PARROT-2 trial demonstrated that serial testing in the same context confers no further benefit.^44^ Since the sFlt1/PlGF ratio test has two components moving in opposite directions in pregnancies complicated by placental MVM disease, the results should be interpreted in the context of the clinical presentation; where a high ratio is driven mostly by hypersecretion of sFlt1, the focus should be on the maternal assessment for preeclampsia with severe features, whereas if a high ratio is driven largely by a low PlGF the focus should be on sonographic evaluation of fetal growth and well-being.^32^ In the context of FGR, PlGF is increasingly being endorsed in clinical practice guidelines to make the important distinction between a constitutionally small for gestational age (SGA) fetus and one with FGR.^45,46^

## Screening for clinical manifestations of placental MVM disease

The use of angiogenic growth factors to screen for placental dysfunction disorders in asymptomatic pregnant individuals must consider the dissociation in time between an early suppression of PlGF and a subsequent excessive production of sFlt1. Both the original description^12^ and more recent publications^13,16^ demonstrate this key point; therefore, PlGF alone, rather than the sFlt1/PlGF ratio, is emerging as a robust screening tool prior for severe placental dysfunction in normotensive individuals prior to fetal viability.^16^ Serial measurement of circulating PlGF levels beginning in the early second trimester in patients at high risk of recurrent placental MVM disease reveals a chronically low pattern,^17^ but, importantly, chronically low PlGF levels precede the subsequent exponential rise in sFlt1 levels that is characteristic of early-onset preeclampsia.^13^ The exemplar of using PlGF as a screening tool is in multimodal (clinical risk factors, PlGF and other biomarkers, and uterine artery Doppler) screening for early-onset preeclampsia at the end of the first trimester, since low-dose aspirin prophylaxis of screen-positive individuals substantially reduces their risk of preterm delivery for this indication.^47^ A summary of potential approaches to aspirin prophylaxis is provided in Table 2. Abnormally low first trimester PlGF, especially in combination with other abnormal biomarkers such as pregnancy-associated plasma protein-A (PAPP-A), is associated with subsequent early preterm birth from preeclampsia or growth restriction.^48,49^ Given the multimodal nature of this first trimester screening approach, considerable reorganization efforts would be needed to deliver these benefits, and seem well-justified based on recent UK^50^ and Canadian^51,52^ reports. Despite endorsement of first trimester screening and prevention care here in Ontario, to date no hospitals have embedded this strategy into their clinical services.

**Table 2.** Risk assessment approaches for aspirin prophylaxis.

Screening approach	Universal aspirin	Clinical risk factors	Multimodal screening
Characteristics	No screen Discontinue based on 24–28 week PlGF? Public health precedent (folic acid)	Age, parity, BMI Medical/OB history	Age, parity, BMI Medical/OB history PlGF Blood pressure Uterine artery doppler
Advantages	True primary prevention	Widely used Inexpensive	Most accurate
Concerns	Equitable? Cost-effective?	Accurate?	Higher cost, resources Feasible?

For patients at high-risk of placental dysfunction who are already receiving aspirin prophylaxis, the combination of PlGF testing and uterine artery Doppler can provide valuable prognostic information on both the risk of early preterm birth, and on the likely underlying placental basis of the associated FGR.^16,17^ In an initial cohort of 102 patients at risk of recurrent placental dysfunction managed at one of our institutions, PlGF levels <10th percentile identified the majority of individuals destined to deliver before 34 + 0 weeks, especially if a second test prior to 20 weeks remained <10th percentile.^53^ A similar evaluation in patients with false-positive first trimester screening results for trisomy 21, due to low circulating PAPP-A demonstrated that PlGF testing in the 15–20 week window was superior to Doppler ultrasound imaging for the identification of a subset destined to require iatrogenic preterm delivery due to preeclampsia and/or FGR.^54^ Larger studies utilizing PlGF testing in these patient populations are therefore awaited as more centers decide to provide PlGF testing to clinicians managing high risk pregnancies. Several PlGF or sFlt1/PlGF ratio assays are now commercially available, including point-of-care tests, the DELFIA Xpress PlGF 1-2-3, and the Elecsys immunoassay; reassuringly all of these assay methods seem to perform similarly the for the primary outcome of predicting the need for delivery with preeclampsia within 14 days of testing.^55^

### Angiogenic factor-directed therapeutic interventions in pregnancy

The ultimate goal of prognostic testing of high-risk patients in the early second trimester is to identify opportunities for interventions beyond aspirin to improve outcomes. These include identifying pregnant individuals that will benefit the most from aspirin prophylaxis, the provision of enhanced education, enhanced sonographic fetal health surveillance, and ultimately access to higher levels of regionalized perinatal care. These established activities may be combined with pharmacologic interventions designed to delay the need for iatrogenic preterm birth and, thus, improve neonatal outcomes. While such interventions are currently in their infancy, one compelling example is the randomized control trial of oral metformin, initiated at admission to provide antihypertensive therapies and enhanced maternal-fetal monitoring for severe preterm preeclampsia; in this context oral metformin was associated with a median 10-day prolongation of gestational age at delivery.^56^ The potential of metformin to suppress preeclampsia has been illustrated in several secondary analyses in clinical trials of pregnant individuals.^57^ Though the actions of metformin in the context of preeclampsia prevention are not fully understood, it appears to reduce sFlt1 production and improve endothelial function.^57–60^

**Figure 2.**
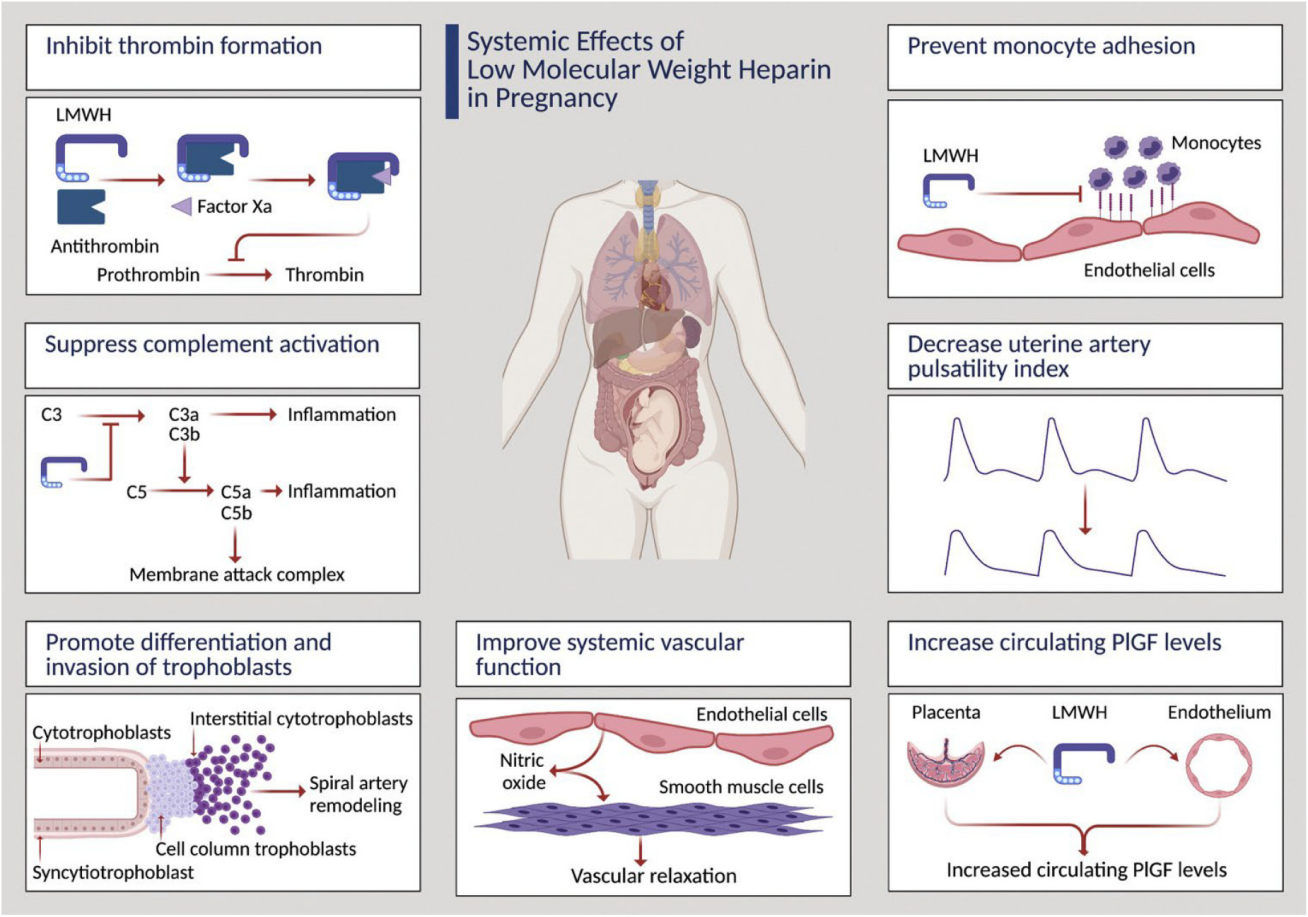
Systemic effects of LMWH in pregnancy.

Source: Reproduced with permission from K. McLaughlin et al. Low-molecular-weight heparin in pregnancies at risk of placental dysfunction. *Am J Obstet Gynecol*. 2022.

The addition of drug interventions to aspirin in the early second trimester, focusing on those at greatest risk of iatrogenic preterm birth, could be strengthened via the concept of attempting to restore deficient PlGF production and release into maternal blood in the early second trimester. In this context, meta-analysis of published randomized trials conducted in patients at high risk of developing preeclampsia comparing aspirin alone or in combination with daily prophylactic injections of low molecular weight heparin (LMWH) indicated that a subset of patients at greatest risk may benefit from LMWH.^61^ Interestingly, LMWH exerts several non-anticoagulant actions within the vasculature that may repress the development of preeclampsia, summarized in Figure 2, that include stimulation of PlGF production both by placental villi^62^ and endothelium.^8^ We have previously shown that normotensive high-risk pregnant individuals with low circulating PlGF in the second trimester may respond acutely to LMWH with a rise in PlGF levels and endothelium-dependent vasorelaxation.^63^ Recently, in a small number of high-risk subjects with serial low PlGF levels between 16–18 weeks of gestation, we observed sustained restoration of deficient PlGF levels associated with pregnancy prolongation and perinatal survival.^9^ These findings are not at odds with two larger negative trials of LMWH in the context of managing pregnant individuals considered to be at high risk of placental dysfunction, because, in both trials, the inclusion criteria were based solely on medical and prior pregnancy risk factors alone^64,65^ and most recruited subjects in one of these trials had normal circulating PlGF levels.^66^ In our experience, a majority of such patients have very favorable outcomes in subsequent pregnancies following an adverse outcome, as they demonstrate normal rising PlGF profiles.^53^ Therefore, a future pilot trial of LMWH could focus on recruiting patients with low circulating PlGF levels in the early second trimester.

An alternative approach may be to suppress circulating sFlt1 levels by directly boosting the actions of progenitor villous cytotrophoblast cells to undertake syncytial fusion and thereby block the aberrant production of sFlt1 by the syncytiotrophoblast later (Figure 2(b)). The drug rosiglitazone, acting as a peroxisome proliferator-activated receptor-Y (PPAR-γ), is capable of boosting syncytialization and thereby repressing sFlt1 in placental villi from pregnancies affected by severe preeclampsia.^67^ These data illustrate the potential of additional drug therapies to prevent preterm delivery from preeclampsia, guided in their design by the identification of those at most risk in the early asymptomatic phase of the disease via low circulating PlGF.

In addition to the development of additional therapeutics, the importance of general health optimization in pregnancy should be acknowledged. Considerations include weight management, smoking cessation, updated vaccinations, vitamin D3 supplementation in winter, and an optimal diet that includes adequate calcium intake. The impact of calcium supplementation in the context of preeclampsia prevention remains unclear. While its potential benefit was supported by a Cochrane review and meta-analysis,^68^ and was subsequently endorsed by the World Health Organization^69^ and other professional societies, more recent work highlights the heterogeneity of evidence, and the over-influence of small trials on the estimated true effect.^70^

### Angiogenic growth factor screening for placenta-mediated pregnancy complications

The most effective screening approach in the first trimester is to incorporate PlGF into a multimodal screening algorithm to direct low-dose aspirin therapy to individual who screen high-risk for developing early-onset preeclampsia.^47^ A simpler method combining PlGF at 16 weeks of gestation with either uterine artery Doppler or a composite of clinical risk factors, provides a similar level of screening precision^16^ and is within the window of effectiveness to commence aspirin prophylaxis.^71^ Note that sFlt1 levels at the end of the first trimester^72^ or the early second trimester^16^ are similar or lower in pregnancies destined to develop early onset preeclampsia compared to healthy pregnancies. Later in the second trimester, PlGF may be combined with placental ultrasound imaging for the prediction of iatrogenic early preterm birth.^73–75^ Systematic review of available studies in 2019 demonstrated that the screening effectiveness of PlGF increases as pregnancy advances closer to the overt manifestations of preeclampsia with severe features or FGR.^76^ Despite lacking any therapeutic intervention at this later stage of pregnancy, the accurate identification of vulnerable pregnant individuals can make possible a number of established interventions, including transfer of care to a regional perinatal center, administration of corticosteroids for fetal lung maturation, and the accurate diagnosis and safe management of preeclampsia with severe features. We recently published a screening study in over 9000 healthy singleton pregnancies at 24–28 weeks of gestation, aligned with screening for gestational diabetes.^77^ Low PlGF (<100 pg/mL) was strongly associated with all early preterm birth <34 weeks, with a positive likelihood ratio of 79.4, and negative likelihood ratio of 0.6. The time to birth was significantly reduced among participants with low PlGF. Furthermore, a positive PlGF screen (<100 pg/mL) identified over 30% of subsequent stillbirths and over 50% of patients subsequently requiring iatrogenic early preterm birth (<34 weeks). Test performance was not influenced by common clinical risk factors (weight, parity, and race) and thus may be used in a simple unimodal fashion. Our findings suggest that PlGF may be a powerful tool to use in countries like Canada that face major geographic challenges for the provision of safe obstetric care.

Midpregnancy angiogenic growth factor testing could help direct not only initiation but also discontinuation of therapy. In the recent StopPRE randomized trial, participants with high-risk first trimester screening, started on aspirin prophylaxis, and subsequent low-risk sFlt1/PlGF (≤38) screening at 24–28 weeks, were randomized 1:1 to discontinuation of aspirin therapy or ongoing treatment, given a concern for aspirin-associated morbidity.^78^ The authors demonstrated non-inferiority to discontinuing aspirin prophylaxis for the primary outcome of preterm preeclampsia. A subsequent post hoc analysis of the StopPRE trial based on screening PlGF alone (normal >100 pg/mL), rather than the sFlt1/PlGF ratio for randomization 1:1 to either discontinuation or ongoing treatment with aspirin^79^ likewise found non-inferiority to this method, compared with continuing aspirin to 36 weeks. These studies reinforce the value of midpregnancy screening to direct third trimester care. Finally, both at the end of the second trimester and later at 36 weeks of gestation, the addition of the sFlt1/PlGF ratio to ultrasound-based screening for suspected FGR demonstrated considerable improvement over ultrasound alone for the prediction of FGR-related morbidity at delivery.^80^

In summary, the availability of PlGF testing, either alone or as the sFlt1/PlGF ratio, has facilitated many improvements in clinical care related to both the accurate diagnosis and screening for the maternal and perinatal placenta-mediated complications of pregnancy. Given the growing body of knowledge related to the underlying molecular placental pathology of both repressed PlGF and hypersecretion of sFlt1, the future holds promise for the development of effective interventions, such as augmentation of systemic VEGF signaling^11^ designed to restore placental function and maternal vascular health, in order to extend pregnancy duration and delivery with improved clinical outcomes.
